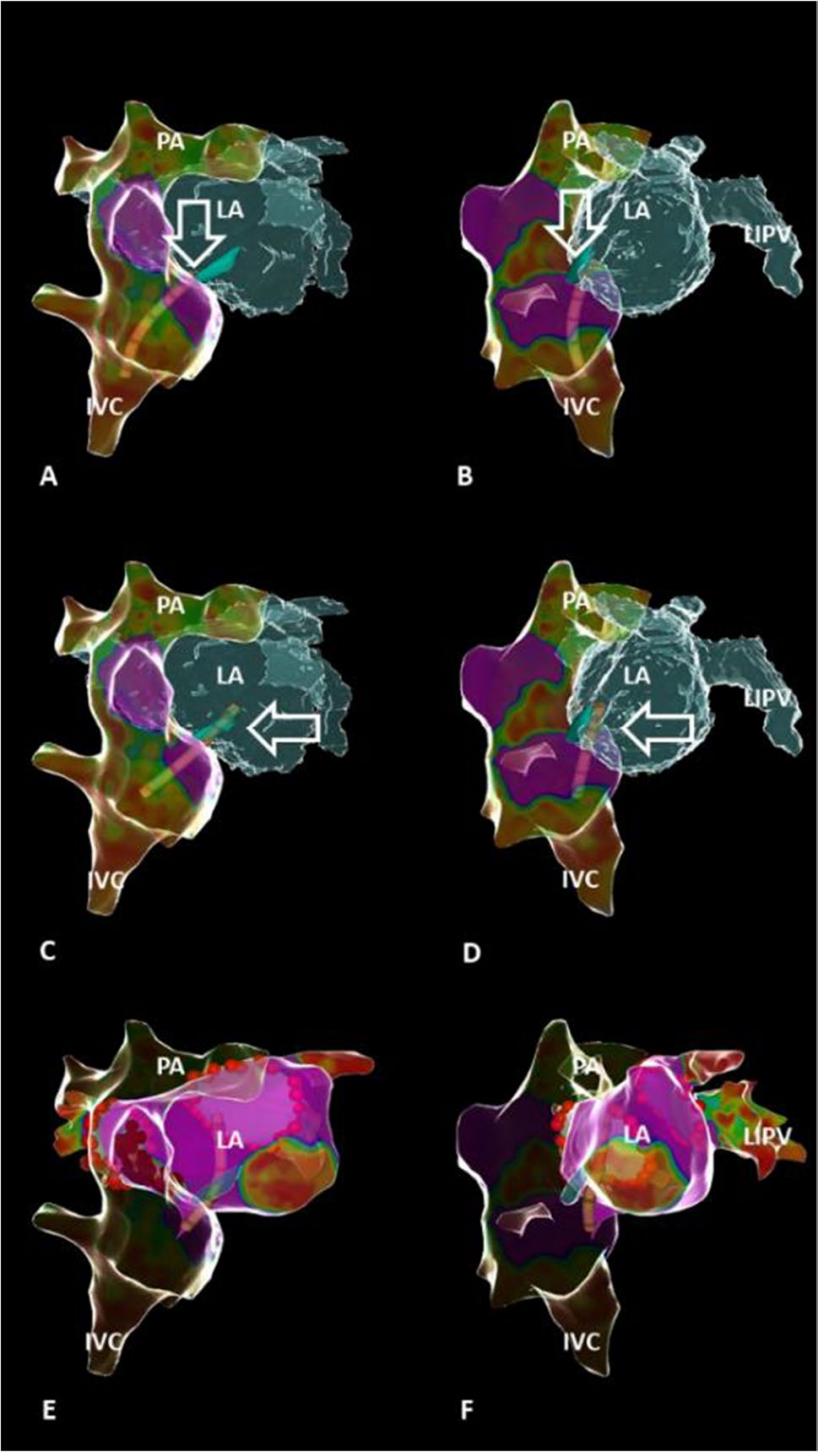# A novel approach for transtunnel puncture in a patient with atrial tachyarrhythmias and Fontan circulation

**DOI:** 10.1007/s10840-021-01047-2

**Published:** 2021-08-24

**Authors:** Christian Sohns, Leonard Bergau, Philipp Sommer, Stephan Molatta

**Affiliations:** 1 Clinic for Electrophysiology, Herz- Und Diabeteszentrum NRW, Ruhr-Universität Bochum, Georgstrasse 11, 32545 Bad Oeynhausen, Germany; 2 Center for Congenital Heart Disease/Pediatric Heart Center, Herz- Und Diabeteszentrum NRW, Ruhr-Universität Bochum, Georgstrasse 11, 32545 Bad Oeynhausen, Germany

In patients with Fontan circulation, lateral intraatrial tunnel, and spontaneously closed lateral tunnel fenestration, transtunnel puncture (TP) might be required to perform ablation in the functional atrium (LA). We report of a patient with drug-refractory atrial arrhythmias and Fontan circulation. This case demonstrates a novel multimodal approach for TP using a steerable sheath (ViziGo, medium curve, Biosense Webster) allowing its visualization inside the 3D-mapping system to avoid the need of fluoroscopy. Intracardiac echography (ICE) was utilized in conjunction with merging the 3D reconstruction from previous magnetic resonance imaging–guided angiography to visualize the complex anatomy in real time and to rule out LA thrombus formation. TP was performed with a Brockenbrough needle BRK1. TP followed a stepwise approach: First, we performed 3D reconstruction of the Fontan tunnel in ***A*** RAO 30° and ***B*** LAO 60° projection. Second, we determined the puncture site (turquoise mark; white arrow) using the visualization of the steerable sheath, a force-sensing catheter (Thermocool SmartTouch SF, Biosense-Webster), and ICE. There was no evidence for a residual fenestration of the tunnel. The sheath was continuously visualized during TP and carefully advanced to the LA (***C*** RAO 30° projection; ***D*** LAO 60° projection). After access to the LA, ***E*, *F*** pulmonary vein isolation was performed with ablation index–guided RF applications (red tags around the ipsilateral pulmonary veins in ***E*, *F***). The clinical atrial tachycardia was induced, mapped, and ablated with substrate modification at the anterior atrial wall in close relationship to the Fontan tunnel (red tags in ***E***; PA, pulmonary arteries; IVC, inferior vena cava; LIPV, left inferior pulmonary vein). There were no procedure-related complications.